# In Vitro Osteogenesis Stimulation via Nano-Hydroxyapatite/Carbon Nanotube Thin Films on Biomedical Stainless Steel

**DOI:** 10.3390/ma11091555

**Published:** 2018-08-29

**Authors:** Natalia M. Martinelli, Maria Julia G. Ribeiro, Ritchelli Ricci, Miller A. Marques, Anderson Oliveira Lobo, Fernanda Roberta Marciano

**Affiliations:** 1 Instituto de Pesquisa e Desenvolvimento, Universidade do Vale do Paraíba, Av. Shishima Hifumi 2911, Bairro Urbanova, São José dos Campos, São Paulo 12244-000, Brazil; natalia_marassi@hotmail.com (N.M.M.); mariajuliagalera1@hotmail.com (M.J.G.R.); ritchelli@gmail.com (R.R.); miller_marques_1993@hotmail.com (M.A.M.); 2 Instituto de Ciência e Tecnologia, Universidade Brasil, Rua Carolina da Fonseca 584, Bairro Itaquera, São Paulo 08230-030, Brazil; lobo.aol@gmail.com

**Keywords:** 316L, electrodeposition, nano-hydroxyapatite, carbon nanotubes, osteoblasts, gene expression

## Abstract

We evaluated the electrophoretic deposition of nanohydroxyapatite/superhydrop hilic multiwalled carbon nanotube composites (nHAp/MWCNT) onto stainless steel biomedical alloys for applications in bone tissue engineering. First, nHAp/MWCNT composites were dispersed into 0.042 mol·L^−1^ of Ca(NO_3_)_2_·4H_2_O + 0.025 mol·L^−1^ NH_4_H_2_PO_4_ electrolytes (pH = 4.8) at two different concentrations. Next, a voltage of −2 V was applied using 316L stainless steel as a working electrode (0.27 cm^2^), a high-purity platinum coil wire was used as the auxiliary electrode, and an Ag/AgCl (3 M) electrode was used as the reference electrode. The nHAp/MWCNT composites were characterized by transmission electron microscopy. The deposited nHAp and nHAp/MWCNT films were characterized by profilometry, scanning electron microscopy, X-ray diffractometry and Raman spectroscopy. Human osteoblast cells were cultivated with the different materials and in vitro cytotoxicity was evaluated using lactate dehydrogenase (LDH) assay. The osteogenesis process was evaluated by mRNA levels of the three genes that are directly related to bone repair: Alkaline Phosphatase, Osteopontin and Osteocalcin. We showed that rough, crystalline apatite thin films containing phases of nHAp were successfully deposited onto 316L stainless steel alloys. Also, we noticed that nHAp/MWCNT thin films deposited onto 316L stainless steel alloys upregulated the expression of important genes related to bone mineralization and maturation. Our results strongly support the possibility of this new alternative to modify the surface of metallic biomedical alloys to promote bone tissue regeneration.

## 1. Introduction

Metallic alloys are the most common metal used to fabricate protheses that promote bone tissue regeneration during the last few decades. Different metal alloys have been used as implants, such as 316L stainless steel [1], Ti6Al4V [2], cobalt alloy [3], titanium, Niquel-Titanium, among others [4]. These have many advantages, including high corrosion resistance, desirable mechanical properties, and partial biocompatibility. Moreover, 316L stainless steel specifically has a lower cost than others, and thus can be an alternative to more people, especially in emergent countries [5,6]; however, none of these materials have been applied to long-term clinical application, especially because they can corrode in biological environments, thereby causing implant failure. To solve problems related to corrosion, chemical modification and deposition of thin micro- and nano-films have been proposed as a solution to this challenge [6,7].

Nanofeature structures are promising because they are similar to the natural components of the extracellular matrix, making them extremely important in the field of bioengineering. Their configurations and physicochemical properties influence the cellular interactions, leading to tissue regeneration, and thus have incredible potential for the development of improved implantable surfaces [8].

Calcium phosphates associated to carbon materials have been widely used in experimental in vitro and in vivo assays to evaluate their potential for use as bone substitutes, due to their excellent biocompatibility, guided bone regeneration, and osteoconductive properties. We recently patented a novel class of nanobiomaterials based on a pioneering method of ultrasound-assisted deposition of nHAp onto superhydrophilic multiwalled carbon nanotube (MWCNT) scaffolds [9]. Furthermore, in a very recent in vitro study, we systematically evaluated the production and characterization of these nanocomposites, focusing on their physical, chemical and biological properties [10]. We showed that nHAp/MWCNT nanocomposites were bioactive and suitable for biomedical applications, with a demonstrated bactericidal effect against *Staphylococcus aureus* (*S. aureus*) and *Escherichia coli* (*E. coli*), with no osteoblast cytotoxicity.

Different techniques have been applied to obtain nHAp thin films with and without the association of carbon nanotubes (CNT) onto biomedical metal alloys. Many alternatives have been applied to obtain thin and homogeneous films onto metallic implants: a shear mixing method [11,12], sprayed plasma [13,14], electrophoretic deposition [15] and electrodeposition [12,16]. Among them, electrodeposition is a simple technique to synthesize HAp/CNT coatings. This method is conducted at a low temperature, allows for good control over the deposition thickness and quality, consumes a low amount of energy, and is an environmentally friendly process.

Herein, a cost-effective and versatile coating technique was applied to obtain nanofeatures onto 316L stainless steel alloys using electrodeposition. A thin and homogeneous high crystalline nHAp/MWCNT composite thin films were electrodeposited onto 316L stainless steel alloys and their chemical, structural and surface properties were evaluated. The in vitro osteogenesis process was also evaluated using human osteoblast cells up to 14 days. The developed nHAp/MWCNT thin films showed superior biological properties, enhancing genes related to mineralization and maturation of human osteoblast cells.

## 2. Materials and Methods

### 2.1. Electrophoretic Deposition

10 × 10 × 1 mm 316L stainless steel samples were polished, cleaned in acetone using ultrasound, and dried under ambient room temperature. nHAp/MWCNT composite and hydroxyapatite were produced as previously reported [17] and characterized by high resolution transmission electron microscopy (FEI-Tecnai G_2_ F20). 0.042 mol·L^−1^ of Ca(NO_3_)_2_·4H_2_O + 0.025 mol·L^−1^ NH_4_H_2_PO_4_ electrolytes (pH = 4.8) were heated at 70 °C. Then, 10 mg·mL^−1^ of two different concentrations of nHAp/MWCNT (1 and 2% of CNT into nHAp matrix) and 10 mg·mL^−1^ of nHAp (1%) were dispersed using an ultrasound (Sonic Vibra-Cell VCX 500, Sonics & Materials, Inc., Newtown, CT, USA) for 30 min. The electrophoretic process was carried out using a classical electrophoretic apparatus (Autolab, PGSTAT 128N, Utrecht, The Netherland). 316L stainless steel alloys were used as working electrodes (0.27 cm^2^), a high-purity platinum coil wire was used as the auxiliary electrode, and an Ag/AgCl (3 M) electrode was used as the reference electrode. The electrochemical parameters were as follows: applied voltage at −5 V and for 7200 s. 

### 2.2. Characterization of nHAp/MWCNT Thin Films

The nHAp crystalline phases were identified using an X-ray diffraction instrument (X-Pert Philips) with Cu Kα radiation (λ = 0.154056 nm), with a 2θ angle of 10° to 50° under the following conditions: voltage of 40 kV, current of 30 mA, step size of 0.02°, and counting time of 2 s per step. The electrodeposited thin film morphology and roughness value were characterized by an optical 3D profilometry (Wyko, Modelo NT 1100, Veeco, Plainview, NY, USA). The diffraction peaks were indexed according to the Joint Committee on Powder Diffraction Standards (JCPDS). The crystal sizes were calculated using Scherrer equation (D_hkl_ = kλ/β_cos(θ)_). The structural analyses of deposited nHAp/MWCNT thin films was identified using Raman spectroscopy (Renishaw, model 2000, Gloucestershire, UK). The spectra were collected after 30 s. The data were plotted using Origin Lab 8^®^. 

### 2.3. Cytotoxicity Test

MG-63 (ATCC^®^ CRL-1427™, Manassas, VA, USA) human osteoblast cell line was used in this study. The culture medium was Dulbecco’s Modified Eagle Medium (DMEM) supplemented with 10% fetal bovine serum (FBS), 100 IU mL^−1^ of penicillin and 100 µg mL^−1^ of streptomycin. Cells were cultured at 37 °C in a 5% CO_2_ humidified incubator. To assess cell viability, we used the LDH assay (TOX7-1KT Sigma, St. Louis, MO, USA). The procedures were done in accordance with manufacturer protocol. The following groups were used: 316L stainless steel, 316L stainless steel covered with (a) nHAp (named nHAp), (b) nHAp/MWCNT_1% (named nHAp/MWCNT_1%), or (c) nHAp/MWCNT_3% (named nHAp/MWCNT_3%), cells (positive control), and DMSO (negative control). Prior to the biological tests, all the samples were sterilized for 24 h under UV irradiation and placed in individual wells of 24-well culture plates. The cells were seeded in each well at a concentration of 2 × 10^5^ cells mL^−1^, supplemented with 10% FBS, with CO_2_ (5%) at 37 °C. After 24 h, 50 μL (1/10 vol %) lysis solution was added and plated for 45 min. Next, 100 μL of LDH were added at each well. After this stage, 150 μL was transferred to a 96-well plate and incubated at room temperature for 30 min (dark). To finalize the reaction, 50 μL of 1 M HCl were added to each well. The optical densities (ODs) were measured at a wavelength of 490 nm and 690 nm (Spectra Count, Packard, Detroit, MI, USA).

The results were analyzed using GraphPad Prism InStat software (version 6.1, San Diego, CA, USA). The ANOVA test (one-way) was used, followed by the Kruskal-Wallis test. Samples with *p* < 0.01 (*n* = 3 in 3 independent experiments) were statistically significant.

### 2.4. Gene Expression Analysis

Reverse transcription-quantitative real-time polymerase chain reaction (RT-qPCR) amplifications evaluate the three genes involved in bone repair: ALPL (alkaline phosphatase), OPN (osteopontin) and OC (osteocalcin). Each experiment was performed in triplicate on an ABI Prism 7500 Sequence Detection System (Applied Biosystems, Foster City, CA, USA) using kit GoTaq^®^ qPCR Master Mix (Promega, São Paulo, Brazil).

Trizol^®^ Reagent (Life Technologies, Rockville, MD, USA) was used for RNA extraction. The 18S and 28S bands were used in 1.5% agarose gel electrophoresis of RNA integrity. Ultraviolet absorption spectroscopy quantified the results using NanoDrop equipment (280/260 and 260/230, ND-1000 Spectrophotometer v.3.0.7—Labtrade). A thermal cycler (Biocycler, MJ96G, Foster City, CA, USA) carried out cDNA synthesis reactions using 2 μg of RNA through a reverse transcription reaction by following the manufacturer’s instructions (ImProm-II^TM^ Reverse Transcription System, Promega, São Paulo, Brazil). The reverse transcription (RT) was performed during 5 min (25 °C), 60 min (42 °C), and 15 min (70 °C). The reaction mixture was stored at −20 °C.

IDT software (Integrated DNA Technologies, www.idtdna.com) and Primer-Blast software (www.ncbi.nlm.nih.gov/tools/primer-blast) determined the primers for amplification of five targets and reference genes. Table 1 lists the used primers. The expression of the reference genes normalized the data. The transcripts of the housekeeping genes *GAPDH* (glyceraldehyde 3-phosphate dehydrogenase), *18SrRNA* (18S ribosomal RNA), and *β-actin* (Actin smooth muscle-beta) and the selected endogenous control *β-actin* gene were quantified. They provided an increased accuracy and resolution in the quantification of gene expression data, which favors the detection of smaller changes in gene expression. Standard PCR conditions were used: 5 min (95 °C), 40 cycles of 15 s (95 °C), 1 min (60 °C), and 5 min (72 °C).

The Relative Delta-Delta Ct (ΔΔCt) method calculated gene expression. The target genes acquired the cycle threshold (Cts) average values, which were compared to the average Cts endogenous gene [18]. The control normalized the results. The obtained results of fold-change are relative to control (Relative Quantification, RQ).

The results of the RT-qPCR were analyzed using the ANOVA (one-way) test, followed by the Dunnett’s multiple-posttest and samples with *p* < 0.05 were considered statistically significant, both in the GraphPad Prisma program version 6.1.

## 3. Results

**Figure 1:**
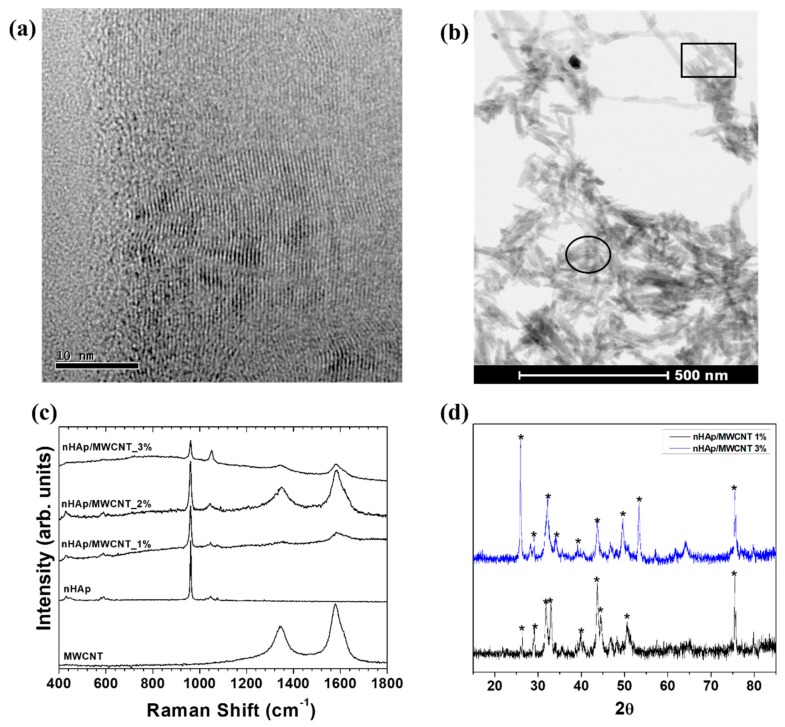
Characterization of MWCNT, nHAp/MWCNT composites and 316L stainless steel alloys covered by nHAp/MWCNT composites. (**a**) High-resolution transmission electron microscopy (HR-TEM) illustrates the internal structure of MWCNT showing the walls. (**b**) Scanning electron microscopy (SEM) identified typical needle-like crystals deposited onto MWCNT. The square illustrates the MWCNT covered by nHAp crystals and the circle illustrates a region containing pure nHAp crystals. (**c**) Raman spectra collected from 316L stainless steel were consistent with those of the nHAp structure, and vibrational modes associated to the carbonate and phosphate phase were identified. The D and G band from the MWCNT are also identified. (**d**) XRD collected from the top of electrodeposited nHAp/MWCNT films. Clearly, the nHAp phase was correctly indexed using a JCPDS card: 024-0033 (identified using *).

The characterization of nHAp/MWCNT before and after electrophoretic process is summarized in Figure 1. The internal structures of MWCNTs are shown in Figure 1a. The tubes have outer diameters around 60 nm and internal wall diameters around 50 nm. The MWCNTs did not have any impurities and had a typical bamboo-like internal structure. The purity is related to the synthesis method and the acid and thermal treatment applied prior to the nanocomposite fabrication [19]. Two different regions were noticed upon analysis of nHAp/MWCNT composites: (a) MWCNT completely covered by nHAp crystals (15 nm in diameter; square region, Figure 1b) and (b) nHAp agglomerates (circle region, Figure 1b). We postulate that the production of nHAp/MWCNT composites is partially homogeneous.

The ultrasound process is a good way to produce nano-ceramic composites, and highly crystalline structures can be produced, especially calcium phosphate structures [20]. Raman spectra collected from all the analyzed samples (Figure 1c) show typical bands attributed to nHAp. Also, we observed the first D and G (1330–1590 cm^−1^) order band from CNTs [21]. The peak at 961 cm^−1^, sharp peak at 1030–1050 cm^−1^, and lower intensity peaks at ~420, 580, and 780 cm^−1^ are typically attributed to crystalline apatite structures, more evident in the nHAp phase [11,22]. Figure 1d shows the X-ray diffraction pattern of the nHAp/MWCNT deposited onto 3l6L stainless steel at two different concentrations. The main peaks attributed to hydroxyapatite were indexed with the JCPDS: 024-0033 card (asterisk). The 002 plane [23] was used to calculate the crystal size using the Scherrer equation (r = 0.89λ/Bcosθ). Each nHAp crystal orientation is specified in the X-ray Diffraction (XRD) pattern, where the main diffraction peaks of HA appear around 28°. The crystal size changed in accordance to the nHAp/MWCNT concentration. When 1% of nHAp/MWCNT composite was used, crystal size was 61.3 nm. In contrast, when 3% was used, a 24.5 nm crystal size was calculated. This result should be noted, and can be correlated directly to the surface roughness measured from the electrodeposited thin films.

**Figure 2:**
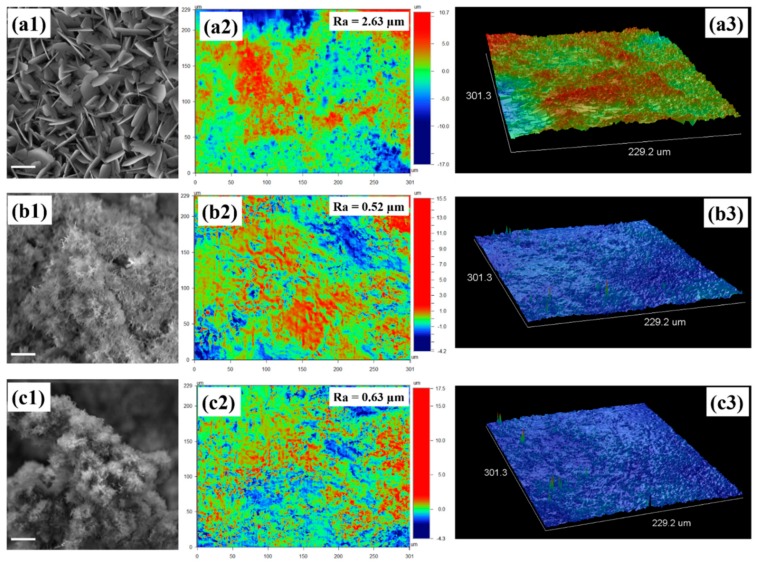
SEM and optical profilometry collected from deposited nHAp and nHAp/MWCNT films on stainless steel alloys. (**a1**) SEM illustrated plate-like crystals of nHAp electrodeposited onto 316L stainless steel alloy; (**a2**) optical images from profilometry collected at the top of nHAp thin films electrodeposited onto 316L stainless steel; (**e3**) 3D constructions extracted from the top of 316L stainless steel alloy; (**b1**) SEM illustrating needle-like crystals electrodeposited onto 316L stainless steel alloy; (**b2**) optical images from profilometry collected at the top of nHAp/MWCNT thin films electrodeposited onto 316L stainless steel; (**c3**) 3D constructions collected from the top of 316L stainless steel alloy illustrating nHAp/MWCNT 1% electrodeposited onto 316L stainless steel; (**c1**) SEM illustrated needle-like crystals electrodeposited onto 316L stainless steel alloy; (**c2**) optical images from profilometry collected at the top of nHAp/MWCNT thin films electrodeposited onto 316L stainless steel; (**c3**) 3D constructions extracted from the top of 316L stainless steel alloy illustrating nHAp/MWCNT 3% elecrodeposited onto 316L stainless steel. SEM scale bars from Figure 2(a1,b1,c1) is 2 µm.

Figure 2(a1–a3) shows the morphology of nHAp deposited onto 316L stainless steel alloys. Typical plate-like crystals were obtained (Figure 2(a1)), which is a common morphology shown by electrodeposited nHAp. A micro-rough film was obtained (Ra = 2.63), as shown in Figure 2(a2). More details about the roughness can be seen from the 3D construction in Figure 2(a3). When nHAp/MWCNT composite (1%) was associated with electrolytic solution, needle-like structures were obtained (Figure 2(b1)). This is more common when a rougher surface is used as the working electrode during the electrophoretic process (specially TiO_2_ nanotubes [13]). Figure 2(b2) illustrates the roughness of obtained thin films. Clearly, we obtained a rougher structure that was 4-fold lower than those obtained by nHAp without MWCNT. More details can be seen from the 3D construction (Figure 2(b3)). This difference in structure can be associated to the presence of carbon nanotubes, which act as growth nuclei for the crystals. A similar crystal morphology was identified when 3% of nHAp/MWCNT composite was used (Figure 2(c1)). The roughness (Figure 2(c2), Ra = 0.63) and 3D (Figure 2(c3)) aspects are practically the same as with 1%. 

**Figure 3:**
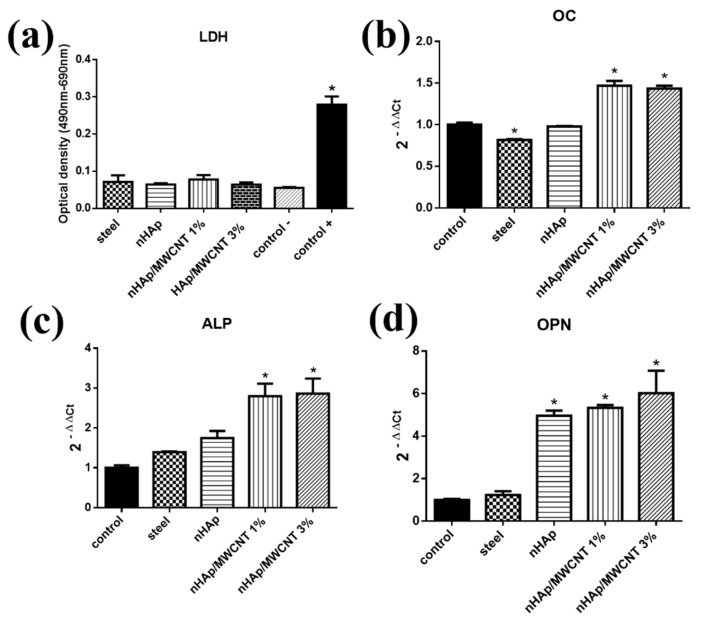
(**a**) Cell viability assay performed by the lactate dehydrogenase (LDH) assay to analyze different groups (316L stainless steel, nHAp, nHAp/MWCNT 1% and nHAp/MWCNT 3%). The control group (−) represents the negative control for cell death, while the control group (+) in which DMSO was added, represents the positive control for cell death. All groups were matched with the control group (−). The mRNA genes (**b**) OC, (**c**) ALP and (**d**) OPN expression of MG-63 lineage cells when cultivated with different analyzed groups were similar to the control. The level of expression of each gene was normalized by the expression of β-actin. The groups with the samples were compared with the control group (cells only). Statistical analysis: LDH—Statistical analysis Oneway ANOVA—Kruskal-Wallis test post * *p* < 0.01. Data from each experiment were obtained in triplicate and are presented as mean ± standard deviation. Gene expression—One-way ANOVA—post-test multiple comparisons Dunnett’s test (* *p* < 0.05). (ALP = Alkaline phosphatase, OPN = Osteopontin, OC = Osteocalcin, nHAp = nano-hydroxyapatite, MWCNT = Carbon Nanotubes).

To the best of our knowledge, this is the first report that nHAp/MWCNT composites were associated with electrolytes, adjusted to Ca/P = 1.67, and a potential was applied to obtain thin films. We also analyzed gene expression related to osteogenic process using these new composites. These findings have important implications for the modification of metal alloys and have great promise for use in biomedical applications. More details about the biological tests are show in Figure 3.

The control group (−, the only group with cells), when compared to the groups of materials (316L, nHAp, nHAp/MWCNT 1% and nHAp/MWCNT 3%), did not present a significant statistical difference (<0.01, Figure 3a). Thus, the assay indicates that the materials (316L, nHAp, nHAp/MWCNT 1% and nHAp/MWCNT 3%) did not produce a cytotoxic effect in the MG-63 strain. In vitro analysis using osteoblasts indicated that materials containing MWCNTs and nHAp demonstrate high proliferation, cell adhesion and had no toxic effects. These results agree with those presented by other authors who have used MWCNTs and HAp with polymeric materials [14,17,24,25,26]. The HAp, as well as the MWCNTs, when used separately, also did not demonstrate cytotoxicity and had an antibacterial effect, thus suggesting great potential for several biomedical applications [27,28].

The expression of genes involved in bone mineralization and maturation in the osteoblastic cells was analyzed when in contact with the materials (316L, nHAp, nHAp/MWCNT 1% and nHAp/MWCNT 3%) after the 14-day period (Figure 3b–d). Figure 3d shows a significant increase in the expression of the OPN gene in the cells of the nHAp/MWCNT 1% and nHAp/MWCNT 3% groups (*p* < 0.01), as well as in the cells of the nHAp group (*p* < 0.05) when compared to the control.

## 4. Discussion

In biomaterial studies, different techniques can be used to measure cell adhesion to understand cell signaling pathways. Cell adhesion could be classified into cell adhesion attachment and detachment events. The attachment events focus on the cell attachment mechanism to the substrate, while the detachment involves the application of the load to detach the adhered cells on the substrate [29]. In order to fully understand how cells behave and function in the human system, both events are equally important and are required [29]. Here, the osteogenesis process was evaluated by the three key genes directly related to bone repair and remodeling.

The osteogenesis process evolves proteins such as alkaline phosphatase (ALP), osteopontin (OPN), osteocalcin (OC), collagen type I (COL-I) and RUNX-2 [30,31]. Briefly, ALP occurs in the early stages of osteogenesis and hydrolyzes organic phosphates, causing phosphorus ions to be released, which are important for the process of extracellular matrix mineralization [32]. OPN is secreted in the early stages of osteoblastic development and mineralization, and acts by binding in the organic and inorganic phase to promote tissue adhesion. The expression of the OPN gene is also associated with increased cell adhesion [30]. OC is an important factor for bone formation and is expressed only by osteoblasts. This protein is translated into the most abundant non-collagenous protein found in bone tissue [33].

It is known that important genes related to osteogenesis can be upregulated on rough surfaces and the extracellular matrix, and mineralization in vitro was enhanced when cultivated onto rough and porous surfaces. From this, we can infer that surface morphology is the most important property to control the morphology and promote osteoblast maturation for increased osteogenesis [8,34,35].

Increased expression of markers such as OPN and OC, indicate an advanced differentiation process and determine osteoblastic maturation and bone mineralization. The expression of the OPN gene is also associated with increased cell adhesion. OC is an important factor for bone formation [36]. This gene is expressed only by osteoblasts, and is translated into the most abundant non-collagenous protein found in bone tissue. An important feature of OC is its affinity with Ca^2+^ and hydroxyapatite [33]. The increased expression of the OC observed in nHAp/MWCNT at 1 and 3% (* *p* < 0.01) are most likely associated both to the affinity of nHAp for the synthesized OC and the increased surface roughness (Figure 2a–c), compared to nHAp alone. The 3D structures highlight (Figure 2a–c) the surface characterization from the nHAp and nHAp/MWCNT composites.

The OPN expression can be positively correlated to the surface roughness of each of the samples, as previously shown in Figure 2(a1–c1). A high expression of the OPN gene occurs in the stage of bone mineralization [37]. Several studies point out that CNTs aid in cell adhesion, which is directly related to the expression of the OPN gene [30,38,39]. The groups containing the HAp/CNT 1%, HAp/CNT 3% samples showed a significant increase in the expression of the alkaline phosphatase (ALP) gene when compared to the control group * *p* > 0.05 (Figure 3c). ALP occurs in the early stages of osteogenesis and plays an important role in bone mineralization. During mineralization, inorganic calcium phosphatase (Ca^2+^) leads to calcification, with subsequent increases of phosphatase in the site, thus participating in the process of bone formation [39]. Osteoblasts participate in the process of bone formation, synthesizing the organic region of the bone matrix and participating in the mineralization of the matrix due to the presence of calcium phosphate. Osteoblasts, when grown in culture, demonstrate peak alkaline phosphatase activity in the 14-day period [28]. The increase of the ALP gene expression in the groups in contact with the nanocomposites, as observed in this work, indicate the potential ability of this material to increase biomineralization of the osteoblasts.

When nHAp/MWCNT composites were used, rougher surfaces were produced, thereby, upregulating osteoblast function. This behavior has been widely reported in the literature [40]. Also, human gingival fibroblast adhesion, proliferation and extracellular matrix deposition have been also reported due to the different structures at micro- and nano-scale levels [41,42,43]. However, in this study we electrodeposited apatite that has a high bioactivity, facilitating more osteoblast adhesion and maturation. The use of nano-hydroxyapatite on the surface of materials implies an increase in alkaline phosphatase expression [39,44]. Other works have also shown that osteoblasts in contact with composites containing CNT and HAp show an increase in ALP expression after a period of 14 days. The overexpression of this gene is probably associated with an increase in the mineralization and matrix protein deposition [37,44]. Gopi et al. dispersed low amounts of CNT to apatite electrolyte and performed electrophoresis. The authors obtained a high adherence that was protective against corrosion. However, the authors only evaluated the in vitro applications using fibroblast cells [12]. Chakraborty et al. [24] recently electrodeposited phosphate coating with and without CNT. The authors showed that the obtained films were bioactive and were able to improve cell adhesion and growth. However, the authors did not investigate specific osteoblast functions, as is presented here.

## 5. Conclusions

A homogeneous and highly crystalline coating onto 316L stainless steel was obtained independent of dispersed nanoparticles. When nHAp/MWCNT composites were used during the electrophoretic process, needle-like crystals were obtained, which produced a rougher coating than those using nHAp alone. All analyzed groups had no cytotoxicity for MG-63 cells. Meanwhile, the presence of nHAp/MWCNT composites upregulated the expression of all analyzed genes related to osteogenesis. Thus, we can conclude that our developed thin films were able to promote in vitro osteoblast maturation and mineralization, a promising first step towards the long-term goal of in vivo application to improve bone repair.

## Figures and Tables

**Table 1:** Details of gene-specific used in RT-qPCR assay.

Gene Symbol/(Access Number)	Gene Name	Primer Sequences	Function
β-actin/ACTB (NM_001101)	Actin Beta	5′-ACCAACTGGGAC GACATGGAGAAA-3′ 5′-TAGCACAGCCTG GATAGCAACGTA-3′	Related to cell motility, structure, and integrity
ALPL (NM_000478.4)	Alkaline phosphatase	5′-CCGTGGCAACT CTATCTTTGG-3′ 5′-GCCATACAGGA TGGCAGTGA-3′	Evolved in bone mineralization
OPN/SPP1 (NM_1251830)	Secreted phosphoprotein 1/Osteopontin	5′-AGACACATAT GATGGCCGAG-3′ 5′-GGCCTTGTATG CACCATTCAA-3′	Specific to cell osteoclast attachment and mineralization of the bone matrix
OC/BGLAP (NM_199173)	Osteocalcin/Bone gamma-carboxyglutamate protein	5′-AAGAGACCCA GGCGCTACCT-3′ 5′-AACTCGTCACA GTCCCGGATTG-3′	Directly secreted by osteoblasts during bone remodeling

## References

[1] Walczak J., Shahgaldi F., Heatley F. (1998). In vivo corrosion of 316l stainless-steel hip implants: Morphology and elemental compositions of corrosion products. Biomaterials.

[2] Grosgogeat B., Reclaru L., Lissac M., Dalard F. (1999). Measurement and evaluation of galvanic corrosion between titanium/Ti6Al4V implants and dental alloys by electrochemical techniques and auger spectrometry. Biomaterials.

[3] Caicedo M.S., Pennekamp P.H., McAllister K., Jacobs J.J., Hallab N.J. (2010). Soluble ions more than particulate cobalt-alloy implant debris induce monocyte costimulatory molecule expression and release of proinflammatory cytokines critical to metal-induced lymphocyte reactivity. J. Biomed. Mater. Res. Part A.

[4] Jacobs J.J., Gilbert J.L., Urban R.M. (1998). Corrosion of metal orthopaedic implants. JBJS.

[5] González J.E.G., Mirza-Rosca J.C. (1999). Study of the corrosion behavior of titanium and some of its alloys for biomedical and dental implant applications. J. Electroanal. Chem..

[6] Hanawa T. (2004). Metal ion release from metal implants. Mater. Sci. Eng. C.

[7] Nanci A., Wuest J.D., Peru L., Brunet P., Sharma V., Zalzal S., McKee M.D. (1998). Chemical modification of titanium surfaces for covalent attachment of biological molecules. J. Biomed. Mater. Res..

[8] Groessner-Schreiber B., Tuan R.S. (1992). Enhanced extracellular matrix production and mineralization by osteoblasts cultured on titanium surfaces in vitro. J. Cell Sci..

[9] Lobo A.O., Marciano F.R., Corat E.J., Trava-Airoldi V.J. (2013). Processo para produção de nanocompósitos de nanoapatitas e os ditos nanocompósitos.

[10] Rodrigues B.V.M., Leite N.C., Cavalcanti B.D.N., da Silva N.S., Marciano F.R., Corat E.J., Webster T.J., Lobo A.O. (2016). Graphene oxide/multi-walled carbon nanotubes as nanofeatured scaffolds for the assisted deposition of nanohydroxyapatite: Characterization and biological evaluation. Int. J. Nanomed..

[11] Koutsopoulos S. (2002). Synthesis and characterization of hydroxyapatite crystals: A review study on the analytical methods. J. Biomed. Mater. Res..

[12] Gopi D., Shinyjoy E., Sekar M., Surendiran M., Kavitha L., Sampath Kumar T.S. (2013). Development of carbon nanotubes reinforced hydroxyapatite composite coatings on titanium by electrodeposition method. Corros. Sci..

[13] Prodana M., Duta M., Ionita D., Bojin D., Stan M.S., Dinischiotu A., Demetrescu I. (2015). A new complex ceramic coating with carbon nanotubes, hydroxyapatite and TiO_2_ nanotubes on ti surface for biomedical applications. Ceram. Int..

[14] Balani K., Anderson R., Laha T., Andara M., Tercero J., Crumpler E., Agarwal A. (2007). Plasma-sprayed carbon nanotube reinforced hydroxyapatite coatings and their interaction with human osteoblasts in vitro. Biomaterials.

[15] Ustundag C.B., Avciata O., Kaya F., Kaya C. (2013). Hydrothermally mixed hydroxyapatite–multiwall carbon nanotubes composite coatings on biomedical alloys by electrophoretic deposition. J. of Phys. Chem. B.

[16] Pei X., Zeng Y., He R., Li Z., Tian L., Wang J., Wan Q., Li X., Bao H. (2014). Single-walled carbon nanotubes/hydroxyapatite coatings on titanium obtained by electrochemical deposition. Appl. Surf. Sci..

[17] Lobo A.O., Zanin H., Siqueira I.A.W.B., Leite N.C.S., Marciano F.R., Corat E.J. (2013). Effect of ultrasound irradiation on the production of nHAp/MWCNT nanocomposites. Mater. Sci. Eng. C.

[18] Pfaffl M.W. (2001). A new mathematical model for relative quantification in real-time RT-PCR. Nucleic Acids Res..

[19] Antunes E.F., de Resende V.G., Mengui U.A., Cunha J.B.M., Corat E.J., Massi M. (2011). Analyses of residual iron in carbon nanotubes produced by camphor/ferrocene pyrolysis and purified by high temperature annealing. Appl. Surf. Sci..

[20] Zou Z., Lin K., Chen L., Chang J. (2012). Ultrafast synthesis and characterization of carbonated hydroxyapatite nanopowders via sonochemistry-assisted microwave process. Ultrason. Sonochem..

[21] Dresselhaus M.S., Dresselhaus G., Saito R., Jorio A. (2005). Raman spectroscopy of carbon nanotubes. Phys. Rep..

[22] Liao S., Xu G., Wang W., Watari F., Cui F., Ramakrishna S., Chan C.K. (2007). Self-assembly of nano-hydroxyapatite on multi-walled carbon nanotubes. Acta Biomater..

[23] Gopi D., Indira J., Prakash V.C.A., Kavitha L. (2009). Spectroscopic characterization of porous nanohydroxyapatite synthesized by a novel amino acid soft solution freezing method. Spectrochim. Acta Part A Mol. Biomol. Spectrosc..

[24] Chakraborty R., Seesala V.S., Sen M., Sengupta S., Dhara S., Saha P., Das K., Das S. (2017). Mwcnt reinforced bone like calcium phosphate—Hydroxyapatite composite coating developed through pulsed electrodeposition with varying amount of apatite phase and crystallinity to promote superior osteoconduction, cytocompatibility and corrosion protection performance compared to bare metallic implant surface. Surf. Coat. Technol..

[25] Lee M., Ku S.H., Ryu J., Park C.B. (2010). Mussel-inspired functionalization of carbon nanotubes for hydroxyapatite mineralization. J. Mater. Chem..

[26] Rodrigues B.V., Silva A.S., Melo G.F., Vasconscellos L.M., Marciano F.R., Lobo A.O. (2016). Influence of low contents of superhydrophilic mwcnt on the properties and cell viability of electrospun poly (butylene adipate-co-terephthalate) fibers. Mater. Sci. Eng. C.

[27] Shi C., Gao J., Wang M., Fu J., Wang D., Zhu Y. (2015). Ultra-trace silver-doped hydroxyapatite with non-cytotoxicity and effective antibacterial activity. Mater. Sci. Eng. C.

[28] Zancanela D.C., Sper Simão A.M., Matsubara E.Y., Rosolen J.M., Ciancaglini P. (2016). Defective multilayer carbon nanotubes increase alkaline phosphatase activity and bone-like nodules in osteoblast cultures. J. Nanosci. Nanotechnol..

[29] Ahmad Khalili A., Ahmad M.R. (2015). A review of cell adhesion studies for biomedical and biological applications. Int. J. Mol. Sci..

[30] Prado R.F.d., de Oliveira F.S., Nascimento R.D., de Vasconcellos L.M.R., Carvalho Y.R., Cairo C.A.A. (2015). Osteoblast response to porous titanium and biomimetic surface: In vitro analysis. Mater. Sci. Eng. C.

[31] Becker J., Lu L., Runge M.B., Zeng H., Yaszemski M.J., Dadsetan M. (2015). Nanocomposite bone scaffolds based on biodegradable polymers and hydroxyapatite. J. Biomed. Mater. Res. Part A.

[32] Bellows C.G., Aubin J.E., Heersche J.N.M. (1993). Differential effects of fluoride during initiation and progression of mineralization of osteoid nodules formed in vitro. J. Bone Miner. Res..

[33] Zhang S., Yang Q., Zhao W., Qiao B., Cui H., Fan J., Li H., Tu X., Jiang D. (2016). In vitro and in vivo biocompatibility and osteogenesis of graphene-reinforced nanohydroxyapatite polyamide66 ternary biocomposite as orthopedic implant material. Int. J. Nanomed..

[34] Lincks J., Boyan B.D., Blanchard C.R., Lohmann C.H., Liu Y., Cochran D.L., Dean D.D., Schwartz Z. (1998). Response of MG63 osteoblast-like cells to titanium and titanium alloy is dependent on surface roughness and composition. Biomaterials.

[35] Bagherifard S., Hickey D.J., de Luca A.C., Malheiro V.N., Markaki A.E., Guagliano M., Webster T.J. (2015). The influence of nanostructured features on bacterial adhesion and bone cell functions on severely shot peened 316l stainless steel. Biomaterials.

[36] Beck G.R., Zerler B., Moran E. (2000). Phosphate is a specific signal for induction of osteopontin gene expression. Proc. Natl. Acad. Sci. USA.

[37] Shao W., He J., Sang F., Ding B., Chen L., Cui S., Li K., Han Q., Tan W. (2016). Coaxial electrospun aligned tussah silk fibroin nanostructured fiber scaffolds embedded with hydroxyapatite–tussah silk fibroin nanoparticles for bone tissue engineering. Mater. Sci. Eng. C.

[38] Sawase T., Jimbo R., Baba K., Shibata Y., Ikeda T., Atsuta M. (2008). Photo-induced hydrophilicity enhances initial cell behavior and early bone apposition. Clin. Oral Implants Res..

[39] Vedakumari W.S., Priya V.M., Sastry T.P. (2014). Deposition of superparamagnetic nanohydroxyapatite on iron–fibrin substrates: Preparation, characterization, cytocompatibility and bioactivity studies. Colloids Surf. B Biointerfaces.

[40] Smith L.L., Niziolek P.J., Haberstroh K.M., Nauman E.A., Webster T.J. (2007). Decreased fibroblast and increased osteoblast adhesion on nanostructured NaOH-etched PLGA scaffolds. Int. J. Nanomed..

[41] Annunziata M., Oliva A., Buosciolo A., Giordano M., Guida A., Guida L. (2012). Bone marrow mesenchymal stem cell response to nano-structured oxidized and turned titanium surfaces. Clin. Oral Implants Res..

[42] Guida L., Oliva A., Basile M.A., Giordano M., Nastri L., Annunziata M. (2013). Human gingival fibroblast functions are stimulated by oxidized nano-structured titanium surfaces. J. Dent..

[43] Calandrelli L., Annunziata M., Della Ragione F., Laurienzo P., Malinconico M., Oliva A. (2010). Development and performance analysis of PCL/silica nanocomposites for bone regeneration. J. Mater. Sci. Mater. Med..

[44] Thakur T., Xavier J.R., Cross L., Jaiswal M.K., Mondragon E., Kaunas R., Gaharwar A.K. (2016). Photocrosslinkable and elastomeric hydrogels for bone regeneration. J. Biomed. Mater. Res. Part A.

